# An anterior mediastinal cystic lesion pathologically confirmed as a mediastinal pancreatic pseudocyst after thoracoscopic resection: a rare case report and literature review

**DOI:** 10.3389/fped.2025.1613764

**Published:** 2025-07-02

**Authors:** Kui Zhai, Xingpeng Miao, Guiling Xue, Zhanyu Yuan, Mao Jia, Mingyan Chen, Renzhong Zha

**Affiliations:** ^1^ Department of Thoracic Surgery, People’s Hospital of Xingyi City, Xingyi, Guizhou, China; ^2^ Department of Pathology, People’s Hospital of Xingyi City, Xingyi, Guizhou, China; ^3^ Department of Price Management, People’s Hospital of Xingyi City, Xingyi, Guizhou, China

**Keywords:** anterior mediastinal cystic mass, pancreatic pseudocyst, mediastinal pancreatic lesion, CT, x-ray

## Abstract

**Background:**

Mediastinal lesions have diverse etiologies, with thymoma, cystic teratoma, and lymphoma being relatively prevalent. In contrast, a pancreatic pseudocyst within the mediastinum is exceedingly rare and can often be mistaken for a thymic cyst or teratoma.

**Case presentation:**

A 17-year-old female presented with a cough and sputum production. Chest CT revealed an anterior mediastinal mass, initially raising the suspicion of a thymic cyst. Thoracoscopic exploration and resection revealed a cystic lesion with a thick wall and brownish fluid. Both frozen section and final histopathological analysis confirmed a mediastinal cyst. Immunohistochemical markers (SYN positive, CK7 positive) led to a diagnosis of mediastinal pancreatic pseudocyst. The patient experienced significant recovery post-surgery, with a marked improvement in symptoms.

**Conclusion:**

This case highlights the importance of including mediastinal pancreatic pseudocyst in the differential diagnosis of anterior mediastinal cystic lesions. A thorough clinical and radiological assessment, along with surgical pathology and immunohistochemical profiling, is essential for accurate diagnosis and appropriate management.

## Introduction

Mediastinal lesions are common yet diagnostically complex conditions encountered in thoracic surgery and respiratory medicine. They may originate from tumors, cysts, infections, or congenital abnormalities involving various tissues or organs. Among mediastinal cystic lesions, thymic cysts, cystic teratomas, pericardial cysts, and bronchogenic cysts are frequently observed. Due to their typical location in the anterior or middle mediastinum, an area densely populated with anatomical structures, and the nonspecific clinical symptoms, diagnostic delays or misdiagnoses are common.

A pancreatic pseudocyst typically arises as a consequence of acute or chronic pancreatitis or pancreatic trauma. It forms from the leakage of pancreatic fluid, tissue necrosis, and inflammatory responses. The pseudocyst wall consists of fibrous or granulation tissue, devoid of an epithelial or glandular lining, distinguishing it from a true cyst (1). Although most pancreatic pseudocysts are located in the retroperitoneum or adjacent to the pancreas, their extension into the thoracic cavity is rare, with isolated cases in the mediastinum being exceedingly uncommon (2, 3). Prior reports have linked mediastinal pancreatic pseudocysts to a history of pancreatitis, abdominal trauma, or pancreatic surgery. However, in some instances, no clear primary pancreatic pathology is identified, and the pathology only reveals an intramediastinal pancreatic pseudocyst, complicating diagnosis (4, 5). Given the wide variety of cystic lesions that can present in the mediastinum and the extremely low incidence of pancreatic pseudocysts in this region, such lesions are frequently misdiagnosed as thymic cysts, cystic teratomas, or lymphoma (6, 7). Moreover, these cystic lesions often manifest with few distinguishing symptoms, and in some cases, patients may only discover them incidentally during routine health checkups. While imaging may suggest a cystic mass, a definitive diagnosis still necessitates pathological examination and immunohistochemical (IHC) analysis. In the current case, a 17-year-old patient without a clear history of pancreatitis or trauma developed a large cystic mass in the anterior mediastinum, which was ultimately resected and confirmed by IHC as a “mediastinal pancreatic pseudocyst”. This case offers valuable insights into diagnostic strategies, pathological evaluation, and treatment approaches, emphasizing the need to consider rare entities in the differential diagnosis of mediastinal cystic lesions.

This case report presents the comprehensive diagnostic and therapeutic course of an anterior mediastinal cystic mass, ultimately diagnosed as a mediastinal pancreatic pseudocyst. By documenting the clinical presentation, imaging findings, surgical procedure, and pathological results, this case underscores the importance of a multidisciplinary approach in managing rare and challenging conditions, offering a reference for early diagnosis and appropriate management of similar cases in the future.

## Case presentation

A 17-year-old female was admitted with a newly detected anterior mediastinal mass, identified one day prior to her admission. Her medical history was unremarkable, and her body weight had remained stable. On detailed questioning, she denied any episodes of acute or chronic abdominal pain, a history of pancreatitis, or abdominal trauma. The day before admission, she visited the outpatient respiratory medicine clinic due to a cough and sputum production. A chest CT scan revealed a cystic lesion located in the anterior and left middle mediastinum, suggestive of a benign tumor, likely a thymic cyst. The patient also reported occasional chest pain, chest tightness, and shortness of breath, though she did not experience fever or chills. For further evaluation and management, she was referred to the thoracic surgery department. Upon admission, her vital signs were: temperature 36.6°C, pulse 90 beats/min, respiration 20 breaths/min, and blood pressure 126/75 mmHg. The patient appeared well-nourished and developed normally, remaining alert and cooperative. No cyanosis or oropharyngeal congestion was observed, and her tonsils were not enlarged. The thoracic cage appeared symmetrical, and percussion revealed resonance. Breath sounds were slightly coarse but absent of rales. Cardiac examination showed a heart rate of 90 beats/min with a regular rhythm and no murmurs. Abdominal examination revealed no tenderness, rebound pain, or organomegaly, and the liver and spleen were not palpable. There was no edema in the lower extremities, and a neurological examination showed normal physiological reflexes without pathological findings.

**Figure 1:**
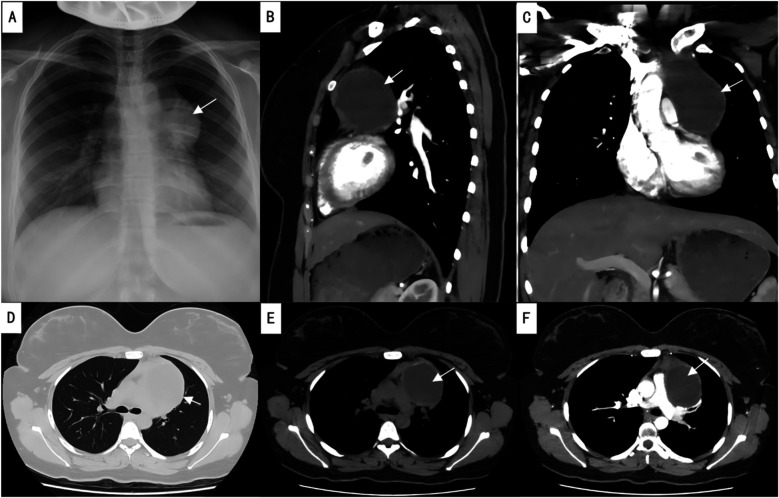
Preoperative imaging studies. **(A)** Preoperative chest x-ray; **(B)** sagittal view of contrast-enhanced chest CT; **(C)** coronal view of contrast-enhanced chest CT; **(D)** lung window of plain chest CT; **(E)** mediastinal window of plain chest CT; **(F)** axial view of contrast-enhanced chest CT. The arrows indicate the location of the lesion.

Imaging findings revealed an enlargement and unclear structure of the left hilum on routine chest x-ray (Figure 1A). Contrast-enhanced chest CT further demonstrated a cystic lesion in both the anterior mediastinum and the left side of the middle mediastinum, suggesting a benign tumor, most likely a thymic cyst, while ruling out a cystic teratoma (Figures 1B–F). Preoperative laboratory tests, including blood work, coagulation function, infectious disease screening, liver and kidney function, and an electrocardiogram, were within normal limits, with no absolute contraindications to surgery identified.

**Figure 2:**
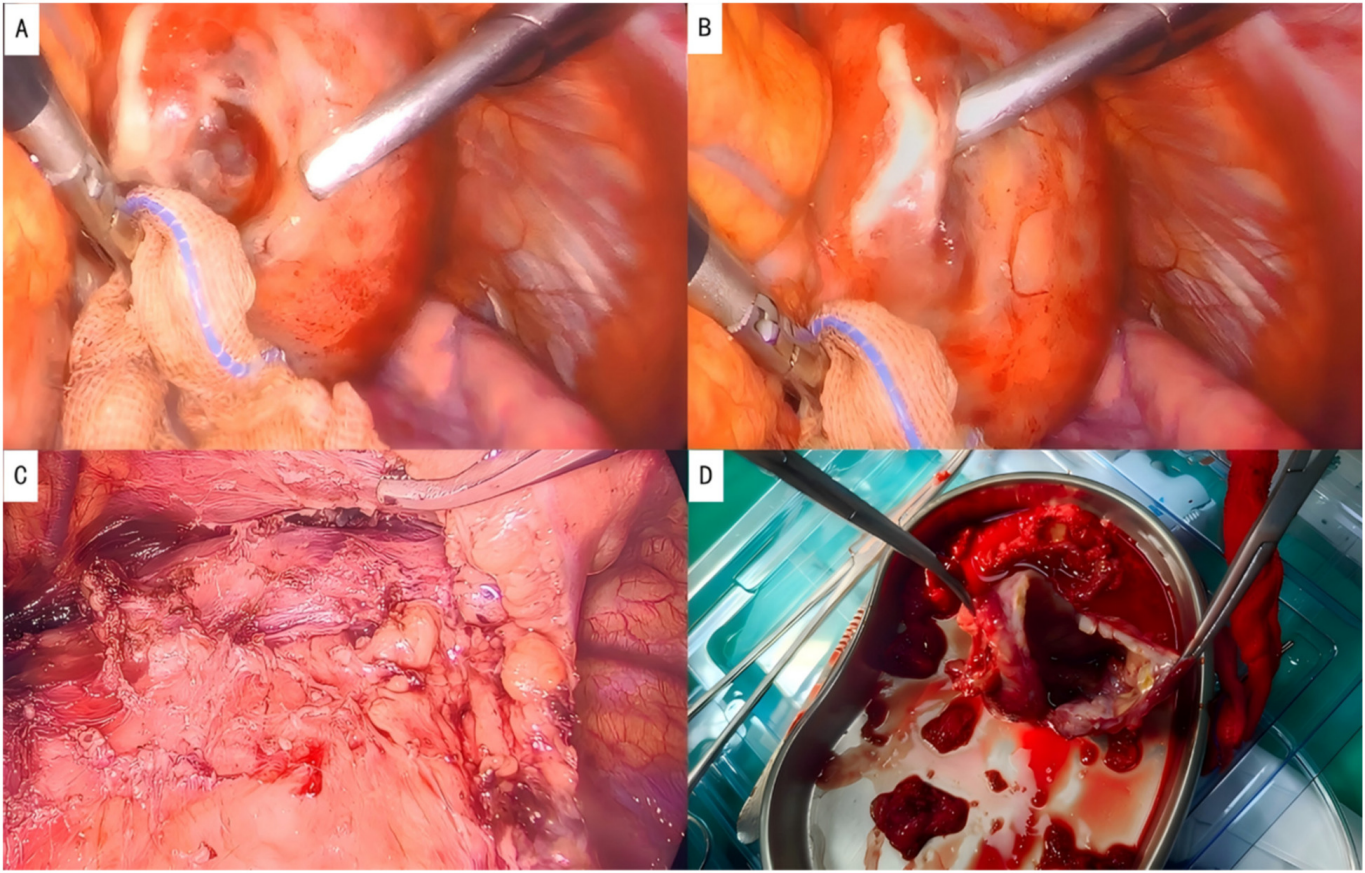
Intraoperative photographs. **(A)** Location of the lesion; **(B)** brown fluid aspirated from the cystic cavity; **(C)** complete resection of the lesion; **(D)** resected specimen, showing a cystic structure.

**Figure 3:**
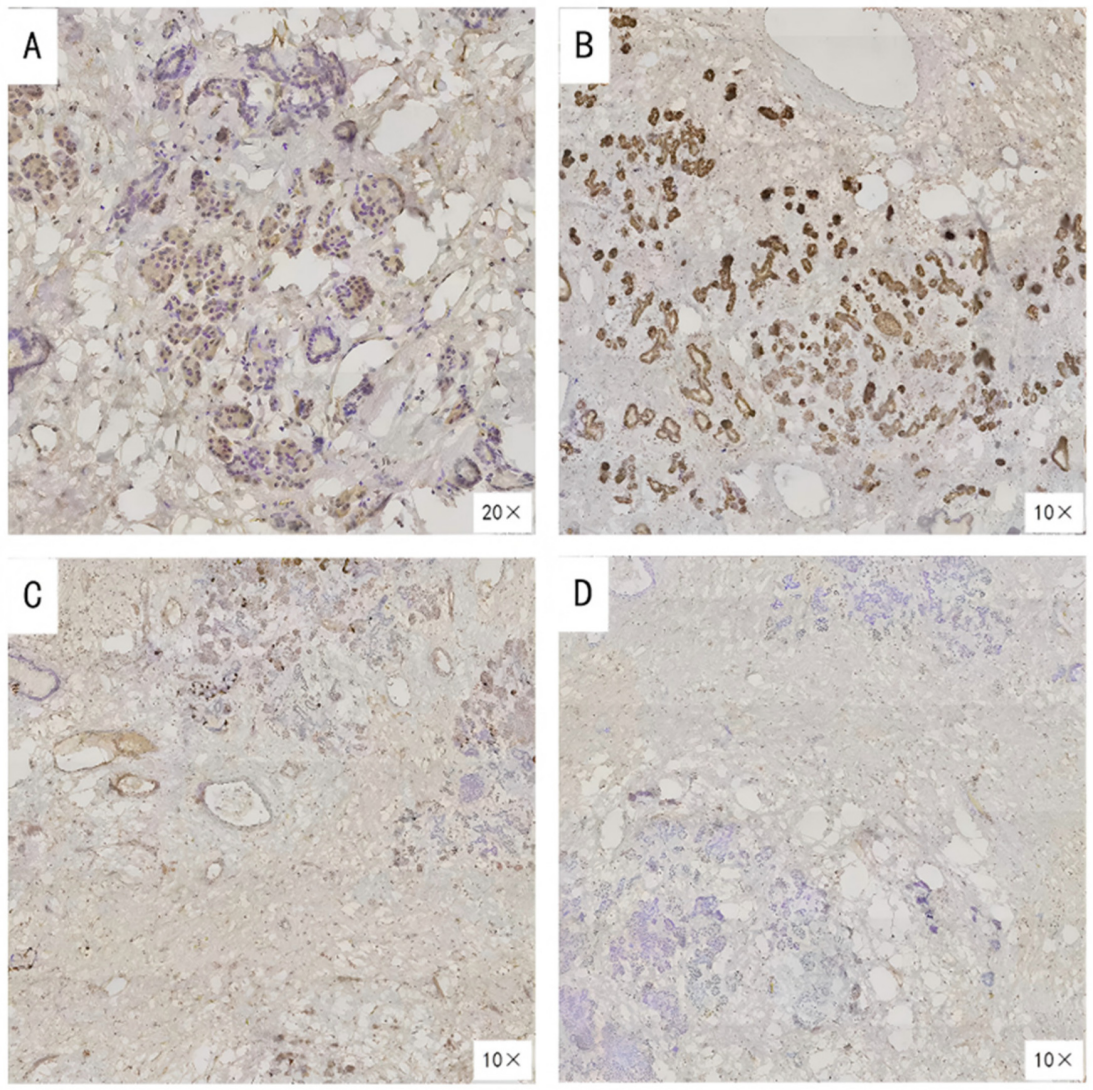
Immunohistochemical findings of the resected lesion. **(A)**SYN (Synaptophysin) positive; **(B)** CK7 (Cytokeratin 7) positive; **(C)** CK20 (Cytokeratin 20) negative; **(D)** MUC-5ac (Mucin 5AC) negative.

**Figure 4:**
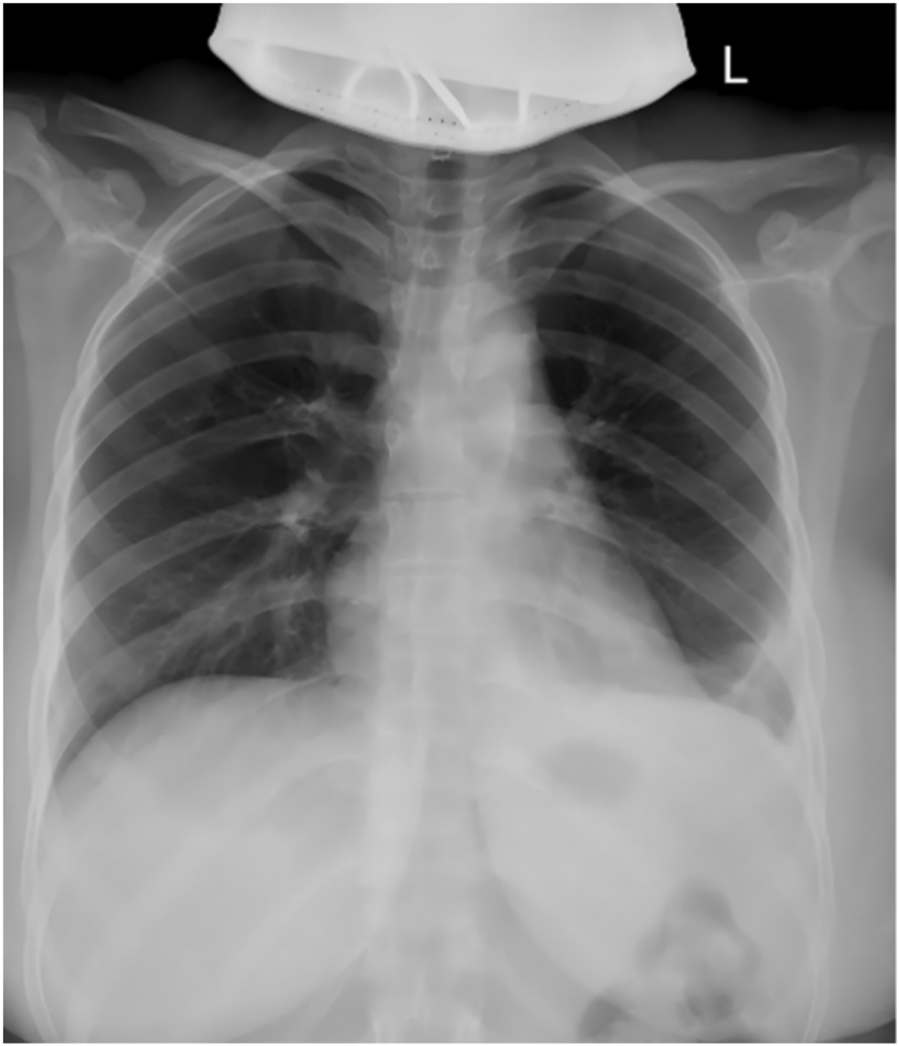
Postoperative follow-up chest x-ray, indicating complete removal of the lesion.

After completing the necessary laboratory tests and confirming the absence of surgical contraindications, the patient underwent “artificial pneumothorax, thoracoscopic resection of the anterior mediastinal tumor via a left thoracic approach, extended thymectomy, and closed chest drainage” under general anesthesia. The procedure involved a 1.5 cm incision at the left mid-axillary line in the 5th intercostal space for a 12 mm thoracoscopic port, with additional operating ports placed at the 5th and 2nd intercostal spaces along the left midclavicular line. After inducing artificial pneumothorax, a cystic lesion measuring 6.5 × 5.0 × 4.5 cm was identified in the anterior mediastinum, partially adhering to the left upper lobe (Figure 2A). Upon opening the cyst wall, brown fluid was released (Figure 2B). The cyst, along with adipose tissue anterior to the pericardium and the retrosternal anterior mediastinum in front of the brachiocephalic vein, was carefully dissected (Figure 2C), and the specimen was retrieved in a bag (Figure 2D). Intraoperative frozen section analysis confirmed the lesion to be a benign cystic structure, and the operation proceeded without complications. Final pathology, along with IHC staining (SYN+, CK7+, CK20–, MUC-5ac–), confirmed the diagnosis of a mediastinal pancreatic pseudocyst (Figures 3A–D). Postoperatively, the patient showed good recovery. A follow-up chest x-ray indicated improvement in the left lung exudative lesion compared to prior images, and her condition remained favorable (Figure 4). The patient was discharged with the diagnosis of “anterior mediastinal cyst (mediastinal pancreatic pseudocyst)”.

## Literature review

A systematic search was conducted across PubMed, Web of Science, EMBASE, the Cochrane Library, and CNKI using the keywords “mediastinal pancreatic pseudocyst”, “pancreatic pseudocyst”, and “mediastinum”, covering publications from January 2004 to April 2025. After removing duplicates and excluding studies lacking full clinical details, seven eligible papers were identified. Including the present case, the series comprised nine cases reported across eight publications (2, 8–13) (Table 1).

**Table 1:** Summary of previously reported mediastinal pancreatic pseudocysts.

Author (year)	Age/sex	Key clinical presentation	Immunohistochemistry	Management & outcome
Sawada et al. (8)	46y/M	Right-sided abdominal pain and dysphagia; chest x-ray showed a mediastinal opacity	Not reported	Improvement after CT-guided percutaneous drainage
Sadat et al. (9)	22y/F	Chest pain, dyspnea, and dysphagia; cyst fluid amylase 18 088 U/L	Not reported	Surgical cystogastrostomy; CT confirmed complete resolution at 3 months
Takimoto and Hara (10)	51y/F	Primary hyperparathyroidism; no chest symptoms; imaging revealed mediastinal cyst	Not reported	Cyst regressed spontaneously after parathyroidectomy
Karamouzos et al. (2)	53y/M	Dyspnea and hemoptysis; large posterior mediastinal cyst with bilateral pleural effusion	Not reported	Initial conservative care → recurrence with bleeding; emergency surgical excision, uneventful recovery
Metaxa et al. (11)	2 cases (38 y M; 40 y M)	Recurrent chest pain/dyspnea; multiple prior episodes of pancreatitis	Not reported	Endoscopic drainage failed; cystogastrostomy/cystojejunostomy performed, symptoms relieved
Blanc and Fusi-Schmidhauser (12)	57y/M	Low-grade fever, dyspnea, and cervical swelling; CT showed cranial extension into the retropharyngeal space	Not reported	Combined percutaneous plus thoraco-abdominal open surgery; complete excision, recovery
Hoang et al. (13)	43y/M	Chest pain and hiatal-hernia–related symptoms; imaging showed cyst traversing esophageal hiatus	Not reported	Supportive medical management and follow-up; gradual cyst shrinkage
Present case (2025)	17y/F	Cough and sputum production; chest CT revealed an anterior mediastinal cystic mass	SYN^+^, CK7^+^, CK20^−^, MUC-5AC^−^	Thoracoscopic complete excision; uneventful follow-up

y, years; M, male; F = female.

The cohort was predominantly male (6/9, 66.7%), with ages ranging from 17 to 57 years (median 46 years). Clinical presentations varied, but respiratory or thoracic symptoms were the most common: five patients reported chest pain and/or dyspnea, two had cough-related complaints, and one presented with massive posterior mediastinal hemorrhage. Only one patient experienced isolated abdominal pain, underscoring the diagnostic challenge posed by ectopic pseudocysts that do not present with typical pancreatitis features.

Management strategies varied based on cyst size, location, and institutional expertise. Three patients underwent minimally invasive drainage or endoscopic procedures; however, two of these patients ultimately required surgery due to persistent or recurrent collections. Primary surgical excision was performed for five patients, including the current case, with three open resections and two thoracoscopic procedures, all resulting in uneventful recoveries. One case report documented the spontaneous regression of a mediastinal pseudocyst following parathyroidectomy for primary hyperparathyroidism.

## Discussion

A 17-year-old female, presenting with cough and sputum production, underwent chest CT, which revealed an anterior mediastinal cystic lesion, prompting hospitalization for further evaluation. The differential diagnosis for mediastinal cystic masses is extensive, including thymic cysts, cystic teratomas, pericardial cysts, bronchogenic cysts, lymphangiomas, and cystic lymph nodes. Thymic cysts typically arise in the anterior mediastinum and present on imaging as well-circumscribed, low-attenuation or fluid-density lesions. While cystic teratomas may also appear predominantly cystic, they typically contain fat or calcifications. Therefore, both non-contrast and contrast-enhanced chest CT are essential for distinguishing solid from cystic lesions and identifying characteristic components, such as fat or calcifications. In this case, the CT showed a cystic mass with no evident fat or calcification, and the patient's clinical presentation was nonspecific, making it challenging to exclude other benign or malignant entities preoperatively, thus necessitating surgical exploration and pathological assessment. Thoracoscopic examination and *en bloc* resection allowed for clarification of the lesion's relationship with adjacent structures and provided an intact specimen for intraoperative frozen section and IHC analysis. The mass, measuring approximately 6.5 × 5.0 × 4.5 cm, was well-demarcated but closely associated with surrounding tissues, and the cyst fluid was brown-tinged. Frozen section suggested a benign cystic process. Routine histology, combined with IHC, confirmed the diagnosis of mediastinal pancreatic pseudocyst, despite the absence of pancreatitis or abdominal trauma in the patient's history. Thus, for unexplained or rare mediastinal cystic lesions, surgery serves both diagnostic and therapeutic roles.

IHC plays a crucial role in diagnosing thoracic tumors and mediastinal masses. In this case, IHC results showed positive staining for SYN (synaptophysin) and CK7 (cytokeratin 7), but negative staining for CK20 (cytokeratin 20) and MUC-5ac (mucin 5AC). SYN is a well-known neuroendocrine marker (14, 15), while CK7 is primarily expressed in ductal epithelia derived from the endoderm, including type II pneumocytes, mammary ductal cells, and some pancreatic ductal cells (16). CK20 is predominantly found in the lower gastrointestinal tract (e.g., colon and rectum) and associated malignancies (17), while MUC-5ac, a member of the mucin family, is typically upregulated in gastric mucosa and certain pancreatic mucinous tumors, making a negative result useful for ruling out typical mucinous tumor components or gastric-type differentiation (18). Given the patient's clinical background and IHC findings, the positive CK7 suggests pancreatic ductal or similar epithelial differentiation, while the negative CK20 excludes lower GI tract or related tumors. The negative MUC-5ac result further rules out prominent mucinous differentiation seen in gastric or pancreatic mucinous lesions. Additionally, SYN positivity indicates neuroendocrine cell involvement in the cyst wall, consistent with pancreatic ductal/islet cell components. These IHC results collectively rule out other common mediastinal cystic lesions, such as thymic epithelium, teratoma, squamous epithelium, and lymphomas. Notably, the patient had no history of pancreatitis, trauma, or surgery, yet developed a pancreatic pseudocyst in the mediastinum, likely due to an occult minor pancreatic duct rupture, ectopic pancreatic tissue, or congenital developmental anomalies. Regardless of the exact mechanism, the IHC findings were instrumental in diagnosing this rare lesion, which was difficult to identify definitively through preoperative imaging (19).

Previous reports reveal significant clinical heterogeneity in patients with mediastinal pancreatic pseudocysts: most presented with chest pain, dyspnea, dysphagia, or cervical swelling, while a minority had pleural effusion or gastrointestinal symptoms. In one case, the lesion was discovered incidentally during an evaluation for primary hyperparathyroidism. In many reports, diagnosis relied on a history of pancreatitis or, more importantly, a markedly elevated amylase level in the cystic fluid—highlighting cyst-fluid amylase measurement as a valuable diagnostic clue. However, only two publications explicitly recorded amylase values, and none provided IHC data. Management strategies ranged from CT-guided or endoscopic drainage to open or minimally invasive surgical excision. Although two patients experienced recurrence with hemorrhage, all published cases eventually achieved favorable outcomes once a definitive diagnosis was established (2, 8–13). In contrast to this historical cohort, thoracoscopic *en bloc* resection in the current case achieved both diagnosis and cure in a single minimally invasive procedure, emphasizing the value of this approach for selected anterior mediastinal cystic lesions (Table 1).

This case offers several important clinical insights. First, rare lesions should be approached with heightened vigilance. While pancreatic pseudocysts are typically found in the retroperitoneal space or near the pancreas, their extension into the mediastinum is uncommon, particularly in the absence of a history of pancreatitis or trauma. This case illustrates that, despite the more frequent occurrence of thymic cysts or teratomas, clinicians should consider a mediastinal pancreatic pseudocyst when confronted with an anterior mediastinal cystic mass. Second, a comprehensive diagnostic approach is essential. While preoperative imaging provides valuable information about the lesion's nature and its relationship with surrounding structures, imaging alone may be insufficient in complex or rare cases. Surgical pathology, combined with IHC, serves as the “gold standard” and is often the most definitive diagnostic tool. In this case, thoracoscopic minimally invasive surgery not only enabled complete resection of the lesion but also facilitated the procurement of reliable pathological specimens. Third, thoracoscopic techniques demonstrate therapeutic feasibility by offering minimal invasiveness, rapid recovery, and excellent maneuverability in diagnosing and treating mediastinal cystic lesions. For suspected benign cystic lesions, early surgical intervention coupled with pathological assessment can promptly alleviate symptoms and exclude potential malignancies, preventing the risk of recurrence or delayed treatment associated with purely conservative management.

Looking ahead, this case can inform future diagnostic and therapeutic strategies for rare mediastinal cystic lesions. First, in addition to definitive histopathology, measurement of cyst-fluid amylase should be considered a critical diagnostic marker, as a markedly elevated level strongly supports a pancreatic origin (20). Second, multimodal imaging techniques—such as endoscopic ultrasound (EUS), MRI, or PET-CT—can provide more detailed information on the cyst wall, internal characteristics, and potential pancreatic lineage, thereby enhancing the accuracy of non-invasive or preoperative assessments. Third, genetic and molecular analyses may help distinguish true pancreatic cysts from pseudocysts and further clarify the pathogenesis of rare pancreatic lesions in the mediastinum. Fourth, ongoing advancements in minimally invasive surgery, particularly thoracoscopy and robotic-assisted techniques, are expected to become the standard for diagnosing and treating anterior mediastinal lesions, offering greater precision, reduced trauma, and faster recovery. Lastly, effective collaboration among thoracic surgeons, gastroenterologists, radiologists, and pathologists remains crucial for early diagnosis and individualized management of these rare mediastinal disorders.

In conclusion, this case emphasizes the need to maintain a broad differential diagnosis for anterior mediastinal cystic lesions, ensuring that rare pathologies are not overlooked. A notable limitation of this case is that cystic fluid was not sampled intraoperatively for amylase measurement. Given the absence of pancreatitis in the patient's history, the working diagnosis was a “thymic cyst”, and the potential diagnostic value of cyst-fluid amylase—highlighted in several previous reports—was not fully considered during surgery. Additionally, since the patient did not present with pancreatic-related symptoms, routine abdominal imaging was not performed, which is another limitation. Although standard imaging can support an initial diagnosis, definitive confirmation relies on surgical specimens and IHC. Minimally invasive thoracoscopic surgery allows for both complete excision of the lesion and ample tissue procurement for pathological examination. As multimodal imaging, molecular diagnostics, and advanced minimally invasive techniques continue to evolve, the outlook for early diagnosis and effective treatment of these rare mediastinal diseases remains promising.

## Data Availability

The original contributions presented in the study are included in the article/Supplementary Material, further inquiries can be directed to the corresponding author/s.
